# Using the UK renal registry for a clinical trial in dialysis patients: the example of SIMPLIFIED

**DOI:** 10.1186/1745-6215-16-S2-O15

**Published:** 2015-11-16

**Authors:** Simon Bond, Rupert Payne, Edward Wilson, Afzal Chowdry, Fergus Caskey, David Wheeler, Thomas Hiemstra

**Affiliations:** 1 Cambridge Clinical Trials Unit, Cambridge, UK; 2 Cambridge University Hospitals NHS Foundation Trust, Cambridge, UK; 3 University of Cambridge, Cambridge, UK; 4 MRC Biostatistics Unit, Cambridge, UK; 5 UK Renal Registry, Bristol, UK; 6 University College London, London, UK

## 

Kidney Failure confers a risk of death that exceeds that of most cancers. Interventions are urgently needed in this patient population. However, kidney failure patients are excluded from almost all interventional trials to avoid confounding, and trial results can therefore not easily be extrapolated. Trials in this population are challenging and expensive. Novel approaches to clinical trials are needed.

Vitamin D deficiency is highly prevalent in dialysis patients and is associated with increased mortality. Its supplementation in dialysis patients is safe.

Native Vitamin D (Cholecalciferol) is a low risk and provides the ideal test bed for novel trials methodologies.

The SIMPLIFIED trial is a large (n=4,200) pragmatic prospective randomised open-label blinded endpoint (PROBE) trial of cholecalciferol versus standard care in patients on dialysis. The primary endpoint is all-cause mortality, and secondary endpoints include CV events, infection, and cancer incidence.

SIMPLIFIED will have no dedicated trial visits, but will capture all data using routine data sources (ONS, HES, UK Renal Registry, PatientView). The UK Renal Registry (UKRR) captures detailed data on all UK dialysis patients, and undertakes linkage with HES and ONS. The UKRR also links with the patient portal PatientView, which allows renal patients to view and update their data.

Patients will consent to their data being directly accessible to the trial team via PatientView.

SIMPLIFIED will be the first UK nephrology trial to use routine data exclusively for trial follow-up, and should help develop expertise and infrastructure to carry out affordable clinical trials within the NHS.

